# Fabrication of freestanding Pt nanowires for use as thermal anemometry probes in turbulence measurements

**DOI:** 10.1038/s41378-021-00255-0

**Published:** 2021-04-02

**Authors:** Hai Le-The, Christian Küchler, Albert van den Berg, Eberhard Bodenschatz, Detlef Lohse, Dominik Krug

**Affiliations:** 1 Physics of Fluids Group, MESA+ Institute, University of Twente, 7522 NB Enschede, The Netherlands; 2 BIOS Lab-on-a-Chip Group, MESA+ Institute, University of Twente, 7522 NB Enschede, The Netherlands; 3 Max Planck-University of Twente Center for Complex Fluid Dynamics, Göttingen, Germany; 4 Max Planck Institute for Dynamics and Self-Organization, 37077 Göttingen, Germany

**Keywords:** Nanosensors, Nanowires

## Abstract

We report a robust fabrication method for patterning freestanding Pt nanowires for use as thermal anemometry probes for small-scale turbulence measurements. Using e-beam lithography, high aspect ratio Pt nanowires (~300 nm width, ~70 µm length, ~100 nm thickness) were patterned on the surface of oxidized silicon (Si) wafers. Combining wet etching processes with dry etching processes, these Pt nanowires were successfully released, rendering them freestanding between two silicon dioxide (SiO_2_) beams supported on Si cantilevers. Moreover, the unique design of the bridge holding the device allowed gentle release of the device without damaging the Pt nanowires. The total fabrication time was minimized by restricting the use of e-beam lithography to the patterning of the Pt nanowires, while standard photolithography was employed for other parts of the devices. We demonstrate that the fabricated sensors are suitable for turbulence measurements when operated in constant-current mode. A robust calibration between the output voltage and the fluid velocity was established over the velocity range from 0.5 to 5 m s^−1^ in a SF_6_ atmosphere at a pressure of 2 bar and a temperature of 21 °C. The sensing signal from the nanowires showed negligible drift over a period of several hours. Moreover, we confirmed that the nanowires can withstand high dynamic pressures by testing them in air at room temperature for velocities up to 55 m s^−1^.

## Introduction

Even today, fully resolved measurements of flow velocities in highly turbulent flows remain highly challenging. The difficulty is best illustrated by considering the nondimensional Reynolds number (*Re*), which measures the turbulence intensity by relating the magnitudes of inertial and viscous forces acting in the flow. Accessing high *Re* flows experimentally is important from a practical perspective, as many engineering applications, such as the boundary layers on the hulls of ships and planes or flow problems in wind farms, fall into this regime. Moreover, measurements in high *Re* flows are also highly relevant to foster and validate our theoretical understanding of turbulence.

A hallmark of turbulence is the fact that “eddying motions,” i.e., seemingly random velocity fluctuations, across a wide range of scales contribute to the evolution of the flow. The range of relevant length scale varies with *Re* as *L/η* ∼ *Re*^3/4^, which renders the measurement challenge obvious^1^. If the largest scale *L* is fixed, e.g., by the size of the lab facilities, then high *Re* can only be reached if the smallest scale *η* (the so-called Kolmogorov scale) is decreased in size. Typical sizes of *η*—and consequently the spatial resolution requirements—are on the order of micrometers. In addition, high temporal resolution is essential to resolve the short turnover timescales of such small eddies^2,3^. Especially in cases where flow structures are advected past the probe by a strong mean flow, such as in investigations of turbulent boundary layers, frequency requirements can reach the order of 100 kHz^4^.

To date, the best resolution and bandwidth characteristics for measuring turbulent velocity fluctuations are achieved using “hot-wire anemometry” (HWA), which is a proven technique with a long history^5–8^. Its measurement principle is based on the velocity-dependent convective cooling of a heated wire element (with wire diameter *d*) placed in the fluid. The time-varying cooling leads to changes in the wire electrical resistance and thus to a voltage signal in the attached electrical circuit, which can be calibrated to yield a fluid velocity measurement. The effective sensor size in HWA is given by the length (*l*) of the wire. However, *l* cannot be decreased arbitrarily because a shorter wire length also increases the portion of the heat that leaves the wire via end conduction, which is unwanted and detrimental to the measurement. This issue can only be overcome if shorter wires are also made thinner. Traditionally, a minimum aspect ratio *l*/*d* ≤ 200 has been used^9^, while more recently, Hultmark et al.^10^ provided a refinement of this criterion. The conventional wire filaments with the best performance characteristics are produced from so-called “Wollaston wires” (thin Pt wires clad in silver) by etching away part of the silver jacket. The sensing element is then formed by the exposed platinum (Pt) wire, for which minimum diameters of ~1 µm can be achieved in this way. Pushing beyond this limit has proven very difficult despite significant efforts. For example, Willmarth and Sharma produced wires with a length of 50 μm using a Wollaston wire 0.5 μm in diameter^11^. However, given the relatively low aspect ratio, the performance of this design was hampered by end-conduction effects. Ligrani and Bradshaw^9^ kept an aspect ratio of ~200 when designing wires with a diameter of 0.625 μm, but with a minimum value of 125 μm, the resulting wire length was still rather large. The need to decrease sensor sizes below this limit initiated a push toward nanofabrication techniques. Early efforts by Löfdahl et al.^12^ yielded only moderate improvements, as their probes featured a large sensing area. Jiang et al.^13^ employed microelectromechanical system (MEMS) techniques to fabricate a polysilicon thermal anemometry probe, but the very good spatial resolution came at the price of significant end-conduction losses in their case. End conduction is also a problem for the multicomponent hot-wire probes (50 μm × 6 μm × 2.7 μm) fabricated by Chen et al.^14^. Moreover, being fixed to a wall, these sensors are not suitable for conventional turbulence measurements.

More recently, the development of a nanoscale thermal anemometry probe, termed NSTAP^15–19^, provided a breakthrough toward unprecedented small-scale resolution. Some noteworthy later developments, such as a microfabricated multiarray probe that provides access to the full velocity gradient tensor^20^ or a specialized hot-wire sensor for measurements in cryogenic helium^21^, have been since reported. For completeness, it should also be mentioned that MEMS techniques have been employed to fabricate small-scale cantilevers for flow measurements^22,23^, but the measurement principle (beam deflection) is different in those cases. In terms of sensor size, the NSTAP remains the state of the art to date. The production process of the NSTAP combines standard photolithography with a series of dry etching and wet etching processes. The sensing element consists of a Pt wire, which is ~100 nm thick, while its width is still 2 μm. The latter arises from a limitation of the photolithography process but in part is also a choice to enhance the convective heat transfer from the wire^16^. Note also that for a variant of the NSTAP, the q-NSTAP reported by Fan et al.^18^, electron-beam lithography is employed. This reduces the width of the wire to between 600 and 800 nm. However, with a length of only 10 µm, the q-NSTAP is designed to measure humidity and is not suited for anemometry. Even with these reduced wire dimensions, the authors reported issues regarding the structural integrity of the sensor due to internal stresses originating from wet etching of silicon dioxide (SiO_2_) to release the wire.

Despite these efforts, the measurement resolution remains the bottleneck for investigations of very high *Re* turbulence in a well-controlled lab environment. In an effort to push the envelope on this, we report a robust method for the fabrication of freestanding Pt nanowires here. These novel wires feature a reduced cross section (300 nm width, 100 nm thickness) compared to existing sensors. The lower cross section offers several advantages. On the one hand, it allows reduction of the effective sensing length while keeping the aspect ratio constraint and thereby limiting conduction losses. Note that with a length of 70 µm, we made a rather conservative choice in the design reported here since as far as fabrication and robustness are concerned, longer wires are more challenging. On the other hand, reducing the cross section also reduces the thermal inertia of the sensor, which will lead to a better frequency response. Moreover, by approaching an aspect ratio of 1 between the width and thickness of the wire, we expect to eliminate spurious angular sensitivity of the measured velocity signal. In this paper, we describe how by combining e-beam lithography (EBL) with wet etching processes and dry etching processes Pt nanowires have been successfully fabricated that are freestanding between two silicon dioxide (SiO_2_) beams supported on Si cantilevers. We further confirm that the fabricated nanowires are capable of and sufficiently robust for measuring the velocity of turbulent flows even at large fluid densities. We tested this in the variable density turbulence tunnel (VDTT) with pressurized SF_6_ as the working fluid as well as in an air flow with velocities up to 55 m s^−1^ without damaging the wires.

## Results and discussion

**Fig. 1:**
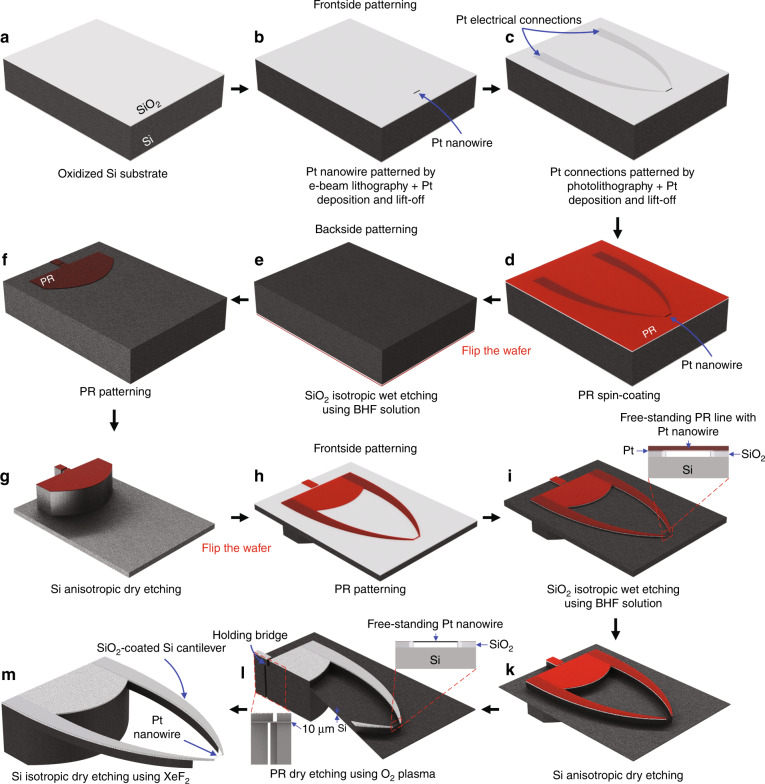
Fabrication process for patterning freestanding Pt nanowires. **a** Wet thermal oxidation of a Si wafer. **b** Patterning of a Pt nanowire using e-beam lithography. **c** Frontside patterning of Pt connections to the Pt nanowire using standard photolithography. **d** Spin-coating of photoresist (PR) on the frontside of the patterned wafer, and **e** wet etching of the SiO_2_ layer on its backside using a BHF solution. **f** Backside patterning of a PR structure of the device base, followed by **g** deep dry etching of Si. **h** Frontside patterning of a PR structure of the support cantilevers. **i** Wet etching of SiO_2_ using a BHF solution, resulting in a freestanding PR line with the Pt nanowire. **k** Dry etching of Si, followed by **l** dry etching of PR using O_2_ plasma at low power. **m** Isotropic dry etching of Si using XeF_2_ for self-release of the device.

Figure 1 presents an overview of the processing sequence for the fabrication of a device featuring a freestanding Pt nanowire. Further details on the dimensions of the structure are provided in the Supplementary information (Fig. S1). We elaborate on individual fabrication steps in the following. Further details and the specific processing parameters employed are provided in the “Materials and methods” section.

### Patterning Pt nanowires using electron-beam lithography

**Fig. 2:**
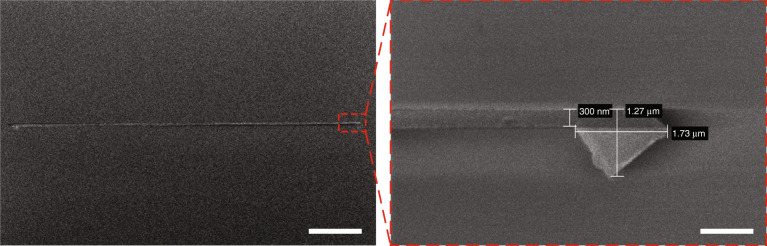
HR-SEM images of a Pt nanowire patterned on the surface of an oxidized Si wafer. Top-view HR-SEM image (scale bar: 10 μm) with a close-up image of the tip of the wire that is expanded slightly to facilitate connection with the Pt micropattern (scale bar: 1 μm).

An EBL system operating at 100 kV (Raith EBPG 5150, Raith GmbH, Germany) was used to pattern Pt nanowires on the surface of oxidized Si wafers (Fig. 1b). These wafers were prepared by wet thermal oxidation of conventional 4-inch (100) silicon (Si) wafers (385 μm thick, Okmetic, Finland) (Fig. 1a). Prior to the sputtering of Pt, a thin titanium (Ti) layer of ~13 nm thickness was sputtered to improve the adhesion of the patterned Pt nanowires. The choice of Ti for the adhesion layer is beneficial here because it can be easily removed together with the SiO_2_ layer in a buffered hydrofluoric (BHF) acid solution, thus leaving freestanding pure Pt nanowires. Figure 2 shows high-resolution scanning electron microscopy (HR-SEM) images of a Pt nanowire fabricated on the surface of an oxidized Si wafer. A well-defined Pt nanowire was obtained with dimensions matching the specifications (~300 nm width, ~70 µm length, ~100 nm thickness). The pattern was expanded slightly at the wire tips to facilitate electrical connection.

### Patterning Pt connections to the Pt nanowires

**Fig. 3:**
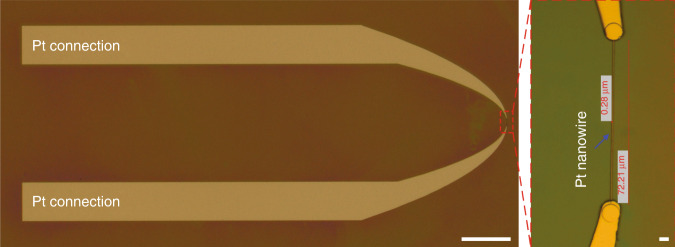
Optical microscopy images of Pt connections. Top-view image (scale bar: 500 μm) with a close-up image of the Pt nanowire location (scale bar: 5 μm).

For electrical connection to the Pt nanowires, Pt micropatterns (termed Pt connections) were fabricated by combining standard photolithography with a lift-off process (Fig. 1c). Figure 3 shows optical microscopy images of Pt connections patterned on the surface of an oxidized Si wafer. It should be noted that the precision of the overlay of the Pt connections with the Pt nanowire is crucial in this step, as any misalignment between these structures can disrupt the electrical connection with the Pt nanowire.

### Backside patterning of the device base using dry etching of Si

Prior to the backside patterning of the wafer, its frontside was covered with a photoresist (PR) layer (Fig. 1d). The wafer was then immersed in a BHF solution to completely remove the SiO_2_ layer on the backside (Fig. 1e), while the SiO_2_ layer on the frontside containing the patterned Pt structures remained protected by the PR coating.

**Fig. 4:**
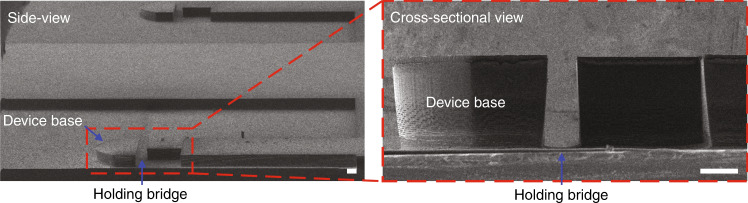
HR-SEM images of backside patterning of the device base using dry etching of Si. Side-viewand cross-sectional HR-SEM images (scale bar: 200 μm).

Subsequently, a PR structure of the device base was patterned on the backside of the wafer using a standard photolithography process (Fig. 1f). The patterned PR structure was hard baked at 120 °C for 10 min to harden the PR areas before conducting etching of Si in an inductively coupled plasma (ICP) deep reactive ion etching (DRIE) instrument (SPTS Pegasus system, UK) using the standard Bosch process (Fig. 1g). Figure 4 shows HR-SEM images of the device base after the dry etching process. It is worth mentioning that a negatively tapered profile was obtained after deep Si etching. This needs to be taken into account when designing the holding bridge for self-release of the device (Fig. 1l).

### Frontside patterning of the device

**Fig. 5:**
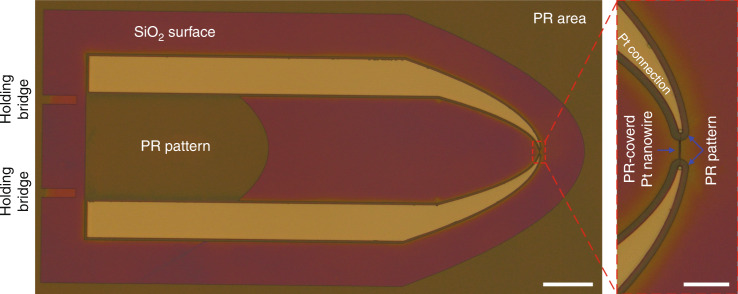
Optical microscopy images of a PR structure patterned on top of the Pt structure. Top-view image (scale bar: 500 μm) with a close-up image of the Pt nanowire location (scale bar: 100 μm).

Figure 5 shows optical microscopy images of a PR structure patterned on top of the Pt structure. The alignment of the patterned PR structure with the Pt structure also needs to be precise in this case so that the PR structure completely covers the Pt structure, especially at the Pt nanowire location where it is covered by a PR line, as shown in the close-up image (Fig. 5). This ensures that the Pt structure is not damaged during the subsequent patterning of the cantilevers by wet etching and dry etching processes (Fig. 1i, k).

**Fig. 6:**
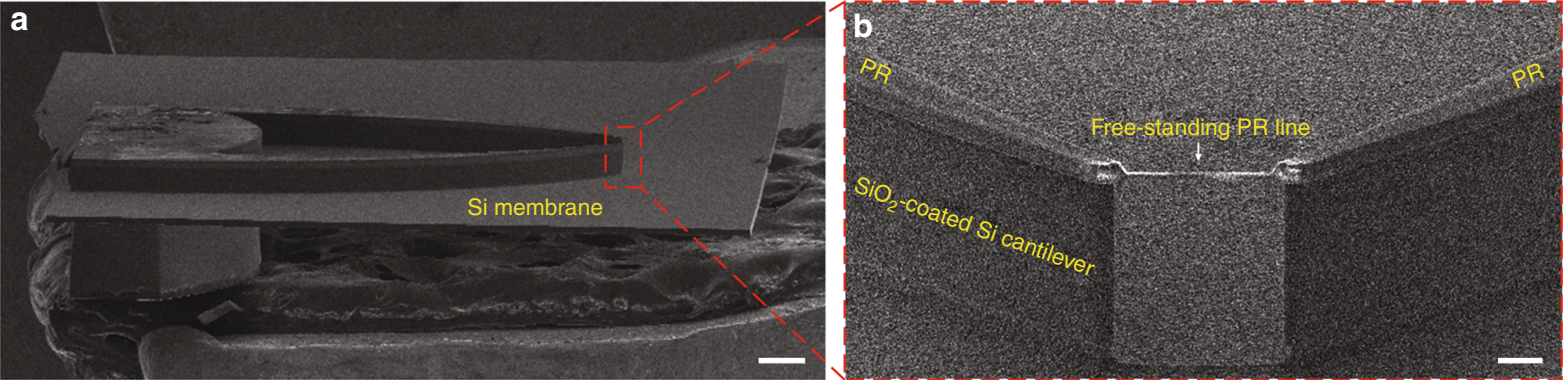
HR-SEM images of support cantilevers after dry etching of Si from the frontside of the wafer. **a** Side-view HR-SEM image (scale bar: 500 µm). Note that the slight damage visible on the top surface of the device base was caused by handling during the SEM inspection. **b** Cross-sectional HR-SEM image (scale bar: 20 µm) of a freestanding PR line with a Pt nanowire.

To release the PR line, the patterned wafers were immersed in a BHF solution for 30 min. As a result, the SiO_2_ under the PR line was etched, thus leaving the freestanding PR line with the Pt nanowire stuck to it (Figs. 1i and 6b). Since both PR and Si are hydrophobic, any liquid trapped between the PR line and the Si surface was quickly and easily removed when spin-drying the wafers. Importantly, this resulted in no damage to the freestanding PR line supporting the Pt nanowire.

Figure 6a shows a side-view HR-SEM image of the support cantilevers after dry etching of Si from the frontside of the wafer. This etching process needs to be stopped when the thickness of the remaining Si membrane (Fig. 1k) is down to ~10 μm. Etching through the Si layer can lead to leakage of cooling gas from the backside, thus terminating the etching process. Crucially, further etching without cooling can result in burning of the Pt line and hence breaking of the Pt nanowire.

To remove the PR covering the Pt nanowire, reactive O_2_ plasma etching was used (Fig. 1l). This needs to be done gently at low power to avoid burning the PR line and thereby breaking the Pt nanowire. The PR removal was conducted before releasing the device because the PR line became brittle after the dry etching process (Fig. 1k). This resulted in frequent damage to the PR line during release, which then also affected the Pt nanowire.

### Isotropic dry etching of Si using XeF_2_

**Fig. 7:**
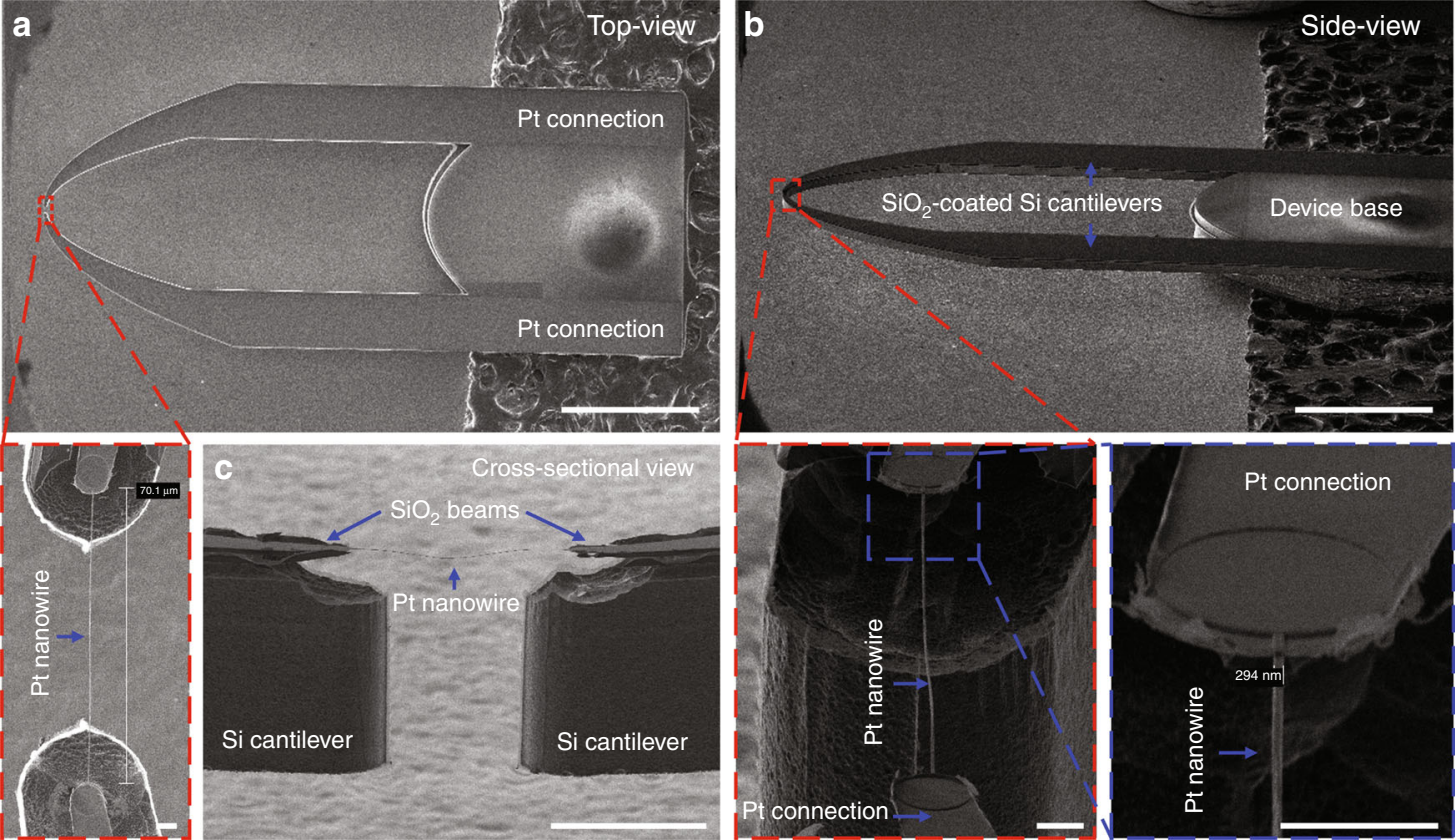
HR-SEM images of a fabricated device consisting of a freestanding Pt nanowire (~300 nm width, ~70 µm length, ~100 nm thickness). **a** Top-view and **b** side-view HR-SEM images (scale bar: 1 mm) with close-up images (scale bar: 5 µm). **c** Cross-sectional HR-SEM image (scale bar: 50 µm) of a fabricated Pt nanowire hanging between two SiO_2_ beams supported on Si cantilevers.

Figure 7 shows HR-SEM images of a fabricated device consisting of a Pt nanowire that is freestanding between two SiO_2_ beams supported on Si cantilevers (Fig. 1m). After isotropic dry etching of Si in XeF_2_, the remaining Si membrane was completely etched, forming two freestanding SiO_2_-coated Si cantilevers (Fig. 7b). It should be noted that the Si underneath the Pt nanowire and the Si at the tip of the two cantilevers were also etched, thus resulting in the Pt nanowire being freestanding on SiO_2_ beams (Fig. 7c).

Owing to the special design of the device holding bridge (Fig. 1l), the final etching step also served to self-release the device from the wafer. The holding bridge also had a remaining Si layer of ~10 μm that was thus etched away in XeF_2_. This self-release procedure has proven necessary and important since it appeared that breaking off the device led to frequent failure of the Pt nanowire (presumably due to vibrations of the cantilevers). As confirmed in the close-up images, the resulting freestanding Pt nanowire has a width of ~300 nm and a length of ~70 μm.

### Batch size and fabrication yield

With our mask design, each 4-inch wafer contains 150 devices. Typical yields in the trial fabrication processes performed thus far were ~50–70% (~70–100 functional devices per wafer). A limiting factor for the fabrication yield was the manual handling of the self-released devices by tweezers after dry etching. We believe that the fabrication yield can be increased further by improving the device handling and by further optimizing the fabrication process steps, especially regarding the uniformity of the dry etching steps over the entire wafer. In addition, it should be mentioned that even thinner wires with widths of 200 and 100 nm were also successfully produced with this process. However, in these cases, the fabrication yield was impractically low, and the wires were not robust enough. We therefore did not pursue the production of wires thinner than 300 nm further.

### Performance of the fabricated devices used as thermal anemometer probes

A typical initial cold resistance for the nanowire was 820 Ω, but this value was observed to drop significantly when the wire was first heated. Annealing the nanowire with incrementally increasing currents up to ~1 mA reduced the resistance to *R*_*w*_ ≈ 740 Ω, and this value was found to be stable over repeated heating cycles with comparable currents. Annealing was performed in the actual experiment with a weak flow of either air or SF_6_ gas. While the cold resistance appeared stable after shorter times, we typically annealed over several hours to avoid any spurious drift in the subsequent tests. By measuring the wire resistance in a temperature-controlled environment, we determined the temperature coefficient of resistivity to be *α*_20_ _ °C_ = 0.0021 K^−1^.

**Fig. 8:**
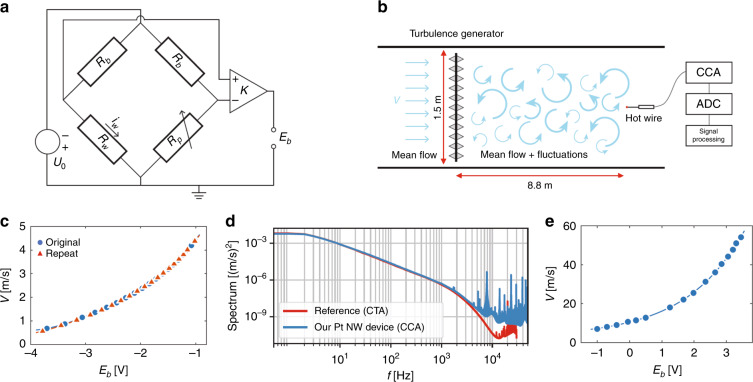
Measurement setup and experiment results of our Pt nanowire device. **a** Sketch of the CCA circuit. **b** Measurement setup in the VDTT. **c** Calibration results in the VDTT along with a repeat taken several hours later. **d** Velocity power spectrum as a function of frequency *f* recorded with our device (in constant-current mode) compared to a commercial reference probe run by a constant-temperature anemometer (CTA). **e** Calibration results in air at room temperature.

The nanowires were operated in a bridge circuit (see Fig. 8a) and tested in the VDTT in Göttingen^24^ in a gaseous sulfur hexafluoride (SF_6_) environment up to extremely high Reynolds numbers (see schematic in Fig. 8b). Note that the purpose of using SF_6_ here is to reduce the kinematic viscosity compared to, e.g., air, which makes it possible to reach high *Re* while keeping the flow velocity moderate. This effect can be enhanced by pressurizing the tunnel up to 15 bar. Further details of the setup and operating conditions are given in the “Materials and methods” section. To calibrate the sensor output voltage *E*_*b*_ against the fluid velocity, a time average of *E*_*b*_ was recorded for several settings of the tunnel velocity *V* in nonturbulent conditions. To gauge the potential drift of the bridge voltage, calibration was performed both before and after a measurement series that spanned several hours. The calibration results are presented in Fig. 8c. There is a clear and monotonic trend between *E*_*b*_ and *V* that can be captured very accurately over the full velocity range by fitting to a fourth-order polynomial (indicated by the lines), which is a standard procedure for hot-wire measurements^25^. Importantly, the calibration results before and after the measurement series are almost indistinguishable, indicating that the drift of the sensor is negligible over an operation period of several hours. As an additional validation, we compare the energy spectra of the fluctuating velocity *v(t)* measured by our probe to results obtained using a standard probe (length 450 µm, diameter 2.5 µm, Dantec Dynamics custom design) as a reference in Fig. 8d. These measurements were taken at 2 bar SF_6_ with a mean flow velocity *V* = 3.75 m s^−1^. The Taylor Reynolds number was *Re*_*λ*_ = 990, and the viscous length scale was *η* ~ 63 µm. Generally, the spectra agree very closely between the two sensors, which is also manifested in the fact that the velocity variances (i.e., the integral of the spectra) differ by only ~1%, which is on the order of the discrepancy expected, as the probes are not located in the exact same location. The collapse of the spectra up to a frequency of *f* ~1 kHz is particularly remarkable since the reference was operated in constant-temperature mode, which offers superior temporal resolution characteristics to the constant-current mode employed to operate our wire here. The slightly elevated noise level at very high frequencies on the order of 10 kHz for our nanowire is a result of the rather basic circuitry and components employed for these first tests. Furthermore, there were no issues operating the wires at pressures up to 15 bar in SF_6_, at which the gas density was more than 1/10th that of water at room temperature. To test whether the wires also perform well in other fluids and at larger flow speeds, we additionally operated the wire in air at room temperature. In this case, the flow was generated by pressurized air exiting a nozzle. Here, the data can also be very well represented by a monotonically increasing fourth-order polynomial across the full range of 5 m s^−1^ ≤ *V* ≤ 55 m s^−1^. The wire was able to withstand the dynamic pressure at the highest velocities without any problems (Fig. 8e).

## Conclusion

In summary, we report a robust fabrication method combining EBL with wet etching and dry etching processes for patterning freestanding Pt nanowires used as thermal anemometer probes for turbulence measurements. With precise control of the dry etching processes, Pt nanowires (~300 nm width, ~100 nm thickness) with a length of 70 µm have been successfully released, rendering them freestanding between two SiO_2_ beams supported on Si cantilevers. A critical aspect is the design of the holding bridge, which ensures a safe and gentle release of the device without damaging the wires. Furthermore, limiting the use of EBL to the patterning of Pt nanowires renders the process cost and time efficient. These benefits far outweigh the additional complications arising from the resulting need to align e-beam and optical lithography patterns with high accuracy. Operational tests have confirmed that the wires are suitable for turbulence measurements in different working media and at high dynamic pressures.

Further characterizations and developments regarding the circuitry, in particular the implementation of a CTA capable of handling relatively high wire resistances, are necessary to exploit the full potential. However, it is already clear that the nanowire design presented here holds much promise regarding several aspects: (1) The more slender wire allows the use of shorter wire lengths without the performance being compromised by end-conduction effects. (2) Smaller sensing elements are expected to improve the frequency response of the anemometer even if the wire is operated in constant-temperature mode^26^. (3) Due to its very small thermal inertia, the wire can yield sufficient frequency resolution for many flow cases even when operated in constant-current mode, as our preliminary results prove here. This eliminates the need for a feedback loop, thereby significantly simplifying the circuitry. (4) The quasi-circular shape of the sensing element is expected to avoid unwanted pitch sensitivity of the sensor. We aim to explore and quantify these benefits in the future in an effort to push the limits for highly resolved high *Re* turbulence measurements.

## Materials and methods

### Wet thermal oxidation of Si wafers

Conventional 4-inch (100) Si wafers (385 μm thick, Okmetic, Finland) with a thick thermal oxide layer of ~2 μm were prepared by wet thermal oxidation (Fig. 1a). Prior to the wet thermal oxidation process, all the Si wafers were cleaned to prevent cross-contamination^27^. Subsequently, the Si wafers were loaded into a high-temperature tube furnace (Model 287, TEMPRESS) using a quartz carrier to implement wet oxidation at 1150 °C for 12 h. During the oxidation process, the flow rate of the mixture of water vapor and nitrogen gas was fixed at 2 l min^−1^. The ramping and cooling rates were set at 10 and 7 °C min^−1^, respectively.

### Patterning Pt nanowires using electron-beam lithography

Prior to e-beam writing, a positive resist (NANO^TM^ 950PMMA Series Resists in Chlorobenzene, MicroChem, USA) was spin-coated over the surface of the oxidized Si wafers at 2500 rpm for 45 s, followed by baking at 165 °C for 2 min. Subsequently, an EBL system operating at 100 kV (Raith EBPG 5150, Raith GmbH, Germany) was used to write the nanowire pattern into the resist layer. The written wafers were then developed in a developer solution (MIBK-IPA mixture) for 90 s, followed by rinsing with deionized (DI) water using a quick dump rinser and spin-drying with nitrogen (N_2_).

A titanium (Ti) layer of ~13 nm and a platinum (Pt) layer of ~100 nm were sputtered over the patterned wafers using an ion-beam sputtering system (home-built T’COathy system, MESA+, NanoLab)^28^. The sputtering processes were performed at 200 W and a pressure of 6.6 × 10^−3^ mbar, which was adjusted using an argon (Ar) flow. Subsequently, the wafers were immersed in acetone with sonication to perform the lift-off process. After rinsing the wafers with DI water and spin-drying with N_2_, the fabrication of Pt nanowires patterned on the surface of the oxidized Si wafers was finished (Fig. 1b).

### Patterning Pt connections to the Pt nanowires

A positive PR layer (OiR 907-17i, Fujifilm, Japan) was spin-coated over the wafer surface at 4000 rpm for 45 s, followed by baking at 95 °C for 1 min. A photomask made of quartz containing inverted chromium (Cr) patterns connected to the patterned Pt nanowires was fabricated in-house by using a mask-making system (DWL 2000 Laser Lithography System, Heidelberg Instruments, Germany). The exposure process was performed by using a mask alignment system (EVG620, EV Group, Austria) for 5 s at an intensity of 12 mW cm^−2^ in hard contact mode. Thereafter, the wafers were post-baked at 120 °C for 1 min, followed by development in an OPD4246 solution for 1 min, rinsing with DI water, and drying with N_2_. A Ti layer of ~6 nm and a Pt layer of ~100 nm were sputtered over the patterned wafers using the T’COathy system. The lift-off process was conducted in acetone with sonication, followed by rinsing the wafers with DI water. After spin-drying with N_2_, the fabrication of Pt connections to the Pt nanowires was completed (Fig. 1c).

### Backside etching of the thermal oxide layer

The patterned surface of the oxidized Si wafers was covered with a PR layer (OiR 908-35, Fujifilm, Japan) by spin-coating at 2000 rpm for 45 s, followed by baking at 95 °C for 3 min (Fig. 1d). The wafers were then immersed in a BHF acid solution for 30 min to completely remove the SiO_2_ layer (etch rate of ~68 nm min^−1^) on their backside (Fig. 1c).

### Backside patterning of the device base using dry etching of Si

After removing the PR layer in acetone, cleaning with DI water, and drying with N_2_ gas, the backside of the wafers was spin-coated with a PR layer (OiR 908-35, Fujifilm, Japan) at 2000 rpm for 45 s, followed by baking at 95 °C for 3 min. A photomask containing a Cr pattern of the device base was used for the exposure process, which was performed by using the mask alignment EVG620 system for 15 s at an intensity of 12 mW cm^−2^ in hard contact mode. Alignment with the frontside Pt structures was performed using bottom alignment in cross-hair mode. Thereafter, the wafers were post-baked at 120 °C for 1 min, followed by development in the OPD4246 solution for 3 min, rinsing with DI water, and drying with N_2_. Subsequently, the wafers were baked at 120 °C for 10 min to harden the remaining PR areas for further backside etching of the Si (Fig. 1f).

The etching of Si was conducted in an ICP DRIE instrument (SPTS Pegasus system, UK) using the standard Bosch process with 105 cycles (0.6 s deposition of C_4_F_8_, 1.75 s etching of Si by SF_6_) (Fig. 1g). After deep Si etching, the wafers were immersed in a 99% nitric acid (HNO_3_) solution for 30 min to completely remove the PR layer and any other residue.

### Frontside patterning of the device

Subsequently, the wafers were flipped, and their frontside was spin-coated with a positive PR layer (OiR 907-17i, Fujifilm, Japan) at 4000 rpm for 45 s, followed by baking at 95 °C for 1 min. A photomask containing a Cr pattern of support cantilevers was used for the exposure process by using the mask alignment EVG620 system for 5 s at an intensity of 12 mW cm^−2^ in hard contact mode. The wafers were then post-baked at 120 °C for 1 min, followed by development in the OPD4246 solution for 1 min, rinsing with DI water, and drying with N_2_. Subsequently, the wafers were baked at 120 °C for 10 min to harden the PR layer (Fig. 1h).

#### Release of the PR line with the Pt nanowire

The patterned wafers were then immersed in the BHF solution for 30 min to completely remove the unprotected SiO_2_ layer. Since the PR line covering the Pt nanowire at the tip of the cantilevers has a small width of ~3 μm, etching in the BHF solution for 30 min resulted in complete removal of SiO_2_ under the PR line and Ti under the Pt nanowire. As a result, the PR line with the Pt nanowire stuck to it was released in this step (Fig. 1i).

#### Patterning support cantilevers using dry etching of Si

The wafers were then etched in the SPTS Pegasus system using the fine etching process with 90 cycles (Fig. 1k) until the remaining Si layer reached a thickness of ~10 µm.

#### Etching of the PR line using O_2_ plasma

To remove the PR covering the Pt nanowire, oxygen (O_2_) plasma etching was performed in a parallel plate reactive ion etching system (home-built TEtske system, MESA+, NanoLab) at the wafer level, 10 mTorr, and 25 W for 20 min. Low-power etching was used to avoid breaking the Pt nanowire during the etching of PR (Fig. 1l).

### Isotropic etching of Si using XeF_2_

For the final patterning of the cantilevers and for release of the devices, the wafers were put in a gas phase Xactix XeF_2_ E1 system (etching time per cycle: 30 s, temperate: 35 °C, pressure: 3000 mTorr) so that the Si was isotropically etched by xenon difluoride (XeF_2_, etching rate of ~1 μm) (Fig. 1m). This resulted in etching through the remaining Si layer, thus forming two freestanding SiO_2_-coated Si cantilevers. The Si underneath the Pt nanowire and the Si at the tip of the two cantilevers were also etched, thus resulting in the Pt nanowire being freestanding on SiO_2_ beams. The device was also self-released after this etching step owing to the special design of the device holding bridge.

### Electrical connection to the device using silver conductive glue

For electrical connection, the fabricated device was mounted on the prongs of a commercial probe holder (Dantec Dynamics A/S, Denmark) using silver conductive glue (Fig. S2). To cure the glue, the device-mounted probe was baked in an oven at 120 °C for at least 15 min.

### Testing the fabricated devices used as thermal anemometer probes

To operate the nanowire, we used a constant-current anemometer (CCA) circuit, as sketched in Fig. 8a. Here, the device was placed in a bridge that features large ballast resistances *R*_*b*_ = 12 kΩ at the top of both arms. Since *R*_*b*_ ≫ *R*_*w*_, this ensures that the wire current *i*_*w*_ remains essentially constant, even as *R*_*w*_ changes slightly. With the nanowire exposed to the flow, we adjust the bridge voltage *U*_*0*_ until the desired overheat ratio *a* = *R*_*w*_ = *R*_*w;*20 °C_ is reached, with typical values of *a* = 1.2–1.4 corresponding to wire overheat temperatures of 100–200 °C. The resistance *R*_*p*_ is chosen such that the bridge is balanced under working conditions. The bridge voltage is then proportional to small differences in *R*_*w*_ that come about as the time-varying cooling by the flow changes the wire temperature slightly. Amplified by a factor *K* = 100 via an instrumentation amplifier, the bridge voltage *E*_*b*_ is recorded as the output parameter of the CCA using an analog-to-digital converter. A calibration and additional signal processing (e.g., filtering) as required finally yield the desired measurement of the fluctuating fluid velocity.

The nanowire was tested in the VDTT in Göttingen described elsewhere^24^. The device was placed in the freestream behind an active turbulence-generating grid, as sketched in Fig. 8b. The grid triggers turbulent motion in the fluid such that the fluid velocity *v(t)* at the hot-wire location fluctuates in time around its mean *V*. For the present set of measurements, the VDTT was operated at a pressure *p* = 2 bar with SF_6_ at a temperature of 21 °C as the working medium. The overheat ratio was set to *a* = 1.24, and the wire current was *i*_*w*_ = 0.622 mA.

## Supplementary information

Supporting Information
