# Supplementary material for: Fabrication of freestanding Pt nanowires for use as thermal anemometry probes in turbulence measurements
Source: Microsyst Nanoeng. 2021 Apr 2;7:28. doi: 10.1038/s41378-021-00255-0 (PMC8433353; doi:10.1038/s41378-021-00255-0)
Supplement: Supplementary file 1 — Supporting Information [file 41378_2021_255_MOESM1_ESM.docx]

**Supporting Information**

**Fabrication of free-standing Pt nanowires for use as thermal anemometry probes in turbulence measurements**

Hai Le-The^1,2,4^, Christian Küechler^3,4^, Albert van den Berg^2,4^, Eberhard Bodenschatz^3,4^, Detlef Lohse^1,4^, Dominik Krug^1.4^

^1^Physics of Fluids Group, MESA+ Institute, University of Twente, Enschede 7522 NB, The Netherlands

^2^BIOS Lab-on-a-Chip Group, MESA+ Institute, University of Twente, Enschede 7522 NB, The Netherlands

^3^Max Planck Institute for Dynamics and Self-Organization, 37077 Göttingen, Germany

^4^Max Planck-University of Twente Center for Complex Fluid Dynamics

Figure S1 Schematic diagram of a fabricated device with detailed dimensions. The fabricated device consists a free-standing Pt nanowire, which is used as a thermal anemometry probe.


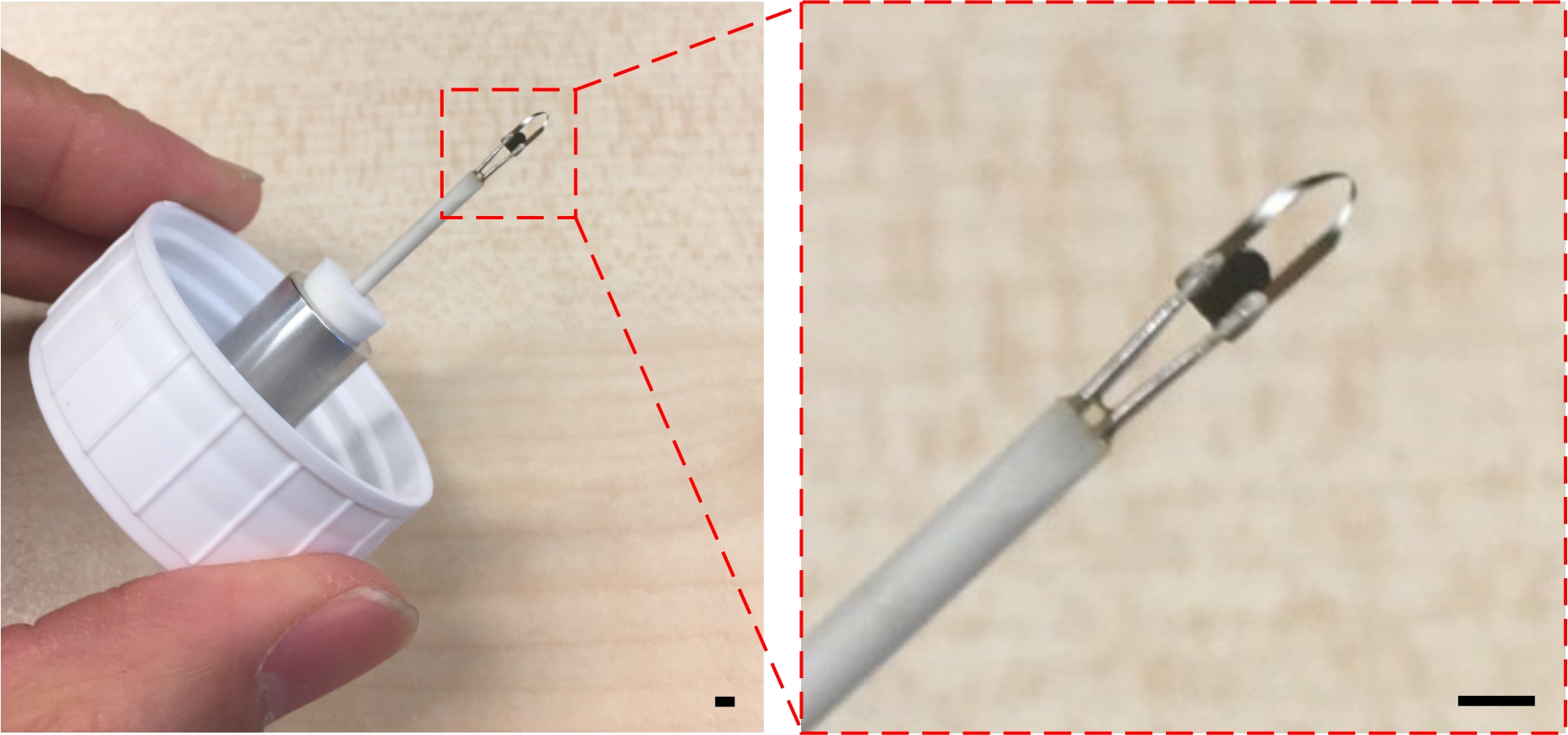


Figure S2 Photographs of a fabricated device mounted on a commercial probe holder, using a silver conductive glue. Scale bars represent 4 mm.
